# Methyl 2-butyl-4-hy­droxy-1,1-dioxo-2*H*-1,2-benzothia­zine-3-carboxyl­ate

**DOI:** 10.1107/S1600536812019733

**Published:** 2012-05-05

**Authors:** Muhammad Nadeem Arshad, Islam Ullah Khan, Muhammad Zia-ur-Rehman, Waseem Ahmed, Abdullah M. Asiri

**Affiliations:** a Department of Chemistry, University of Gujrat, Gujrat 50781, Pakistan; b Materials Chemistry Laboratory, Department of Chemistry, GC University, Lahore 54000, Pakistan; c Applied Chemistry Research Centre, PCSIR Laboratories Complex, Lahore 54600, Pakistan; d Department of Biochemistry, Federal Urdu University of Arts Science and Technology, Gulshan-e-Iqbal Campus, Karachi, Pakistan; e The Center of Excellence for Advanced Materials Research, King Abdul Aziz University, Jeddah, PO Box 80203, Saudi Arabia

## Abstract

In the title compound, C_14_H_17_NO_5_S, the thia­zine ring adopts a half-chair conformation. The mol­ecule exhibits an intra­molecular O—H⋯O hydrogen bond, which forms a six-membered *S*(6) ring motif. The planes of the benzene and thia­zine rings are inclined at a dihedral angle of 15.30 (12)°.

## Related literature

For the synthesis, see: Arshad *et al.* (2011*a* ▶). For biological activity of related compounds, see: Zia-ur-Rehman *et al.* (2006 ▶). For related structures, see: Arshad *et al.* (2011*b* ▶, 2012 ▶); For graph-set notation, see: Bernstein *et al.* (1995 ▶). For puckering parameters, see: Cremer & Pople (1975 ▶).
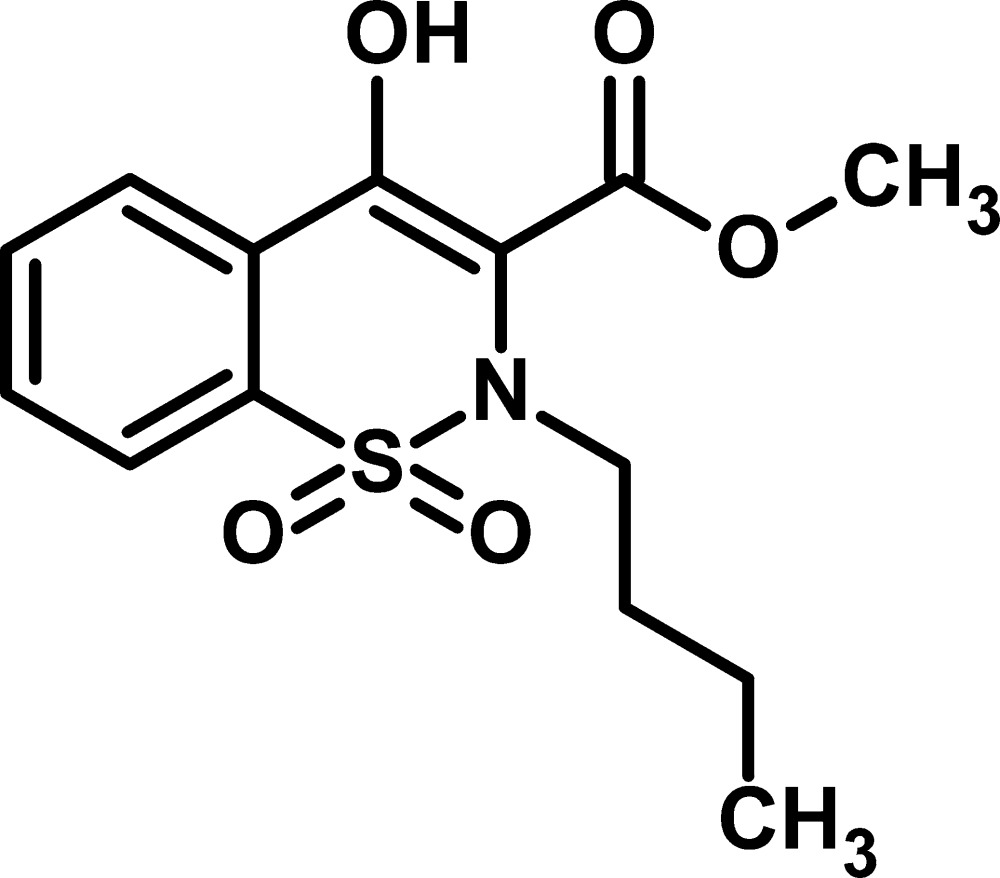


## Experimental

### 

#### Crystal data

C_14_H_17_NO_5_S
*M* *_r_* = 311.35
Monoclinic, 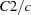

*a* = 25.173 (7) Å
*b* = 9.280 (2) Å
*c* = 12.531 (3) Å
β = 91.741 (3)°
*V* = 2926.0 (13) Å^3^
*Z* = 8
Mo *K*α radiation
μ = 0.24 mm^−1^
*T* = 100 K
0.44 × 0.31 × 0.25 mm

#### Data collection

Bruker SMART 1K diffractometer
Absorption correction: multi-scan (*SADABS*; Bruker, 2001 ▶) *T*_min_ = 0.901, *T*_max_ = 0.942
12490 measured reflections
3498 independent reflections
3132 reflections with *I* > 2σ(*I*)
*R*_int_ = 0.031

#### Refinement

*R*[*F* ^2^ > 2σ(*F* ^2^)] = 0.070
*wR*(*F* ^2^) = 0.210
*S* = 1.10
3498 reflections
193 parameters
H-atom parameters constrained
Δρ_max_ = 1.70 e Å^−3^
Δρ_min_ = −0.49 e Å^−3^

### 

Data collection: *SMART* (Bruker, 2001 ▶); cell refinement: *SAINT* (Bruker, 2001 ▶); data reduction: *SAINT*; program(s) used to solve structure: *SHELXS97* (Sheldrick, 2008 ▶); program(s) used to refine structure: *SHELXL97* (Sheldrick, 2008 ▶); molecular graphics: *PLATON* (Spek, 2009 ▶) and *X-SEED* (Barbour, 2001 ▶); software used to prepare material for publication: *WinGX* (Farrugia, 1999 ▶) and *PLATON*.

## Supplementary Material

Crystal structure: contains datablock(s) I, global. DOI: 10.1107/S1600536812019733/im2370sup1.cif

Structure factors: contains datablock(s) I. DOI: 10.1107/S1600536812019733/im2370Isup2.hkl

Supplementary material file. DOI: 10.1107/S1600536812019733/im2370Isup3.cml

Additional supplementary materials:  crystallographic information; 3D view; checkCIF report

## Figures and Tables

**Table 1:** Hydrogen-bond geometry (Å, °)

*D*—H⋯*A*	*D*—H	H⋯*A*	*D*⋯*A*	*D*—H⋯*A*
O1—H1*O*⋯O4	0.84	1.85	2.564 (4)	142

**Fig. 1.**
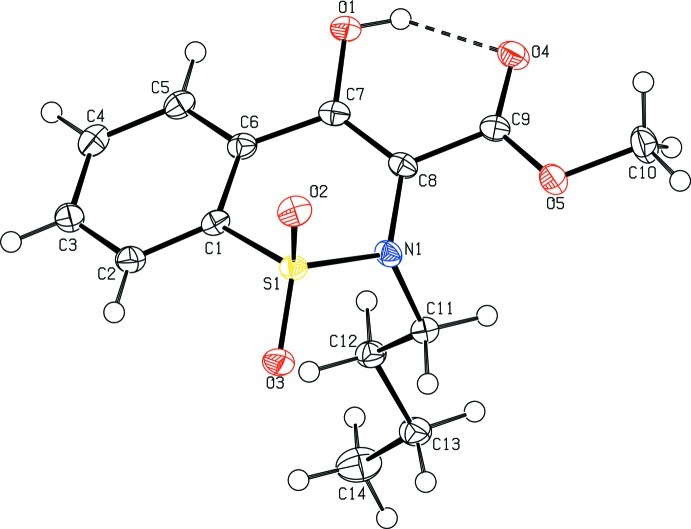
ORTEP diagram of the molecular structure of (I) showing intramolecular O—H···O hydrogen bonding as a dashed line and thermal ellipsoids at the 50% probability level.

## supplementary crystallographic information

### Comment

Our research group already reported the synthesis and biological activities (Arshad *et al.*, 2011*a*; Zia-ur-Rehman *et al.*, 2006) of the title compound as well as the crystal structures of related compounds (Arshad *et al.*, 2011*b*, 2012).

The title compound is the *N*-butyl derivative of methyl-4-hydroxy-1,1-dioxo-2*H*-1,2-benzothiazine-3-carboxylate. The methyl ester moiety attached to the thiazine ring shows an almost planar geometry with a root mean square (r. m. s.) deviation of 0.0034 (14) Å and is oriented at a dihedral angle of 11.4 (2)° and 10.5 (2)° with respect to the thiazine (C1/C6/C7/C8/N1/S1) and aromatic benzene (C1/C2/C3/C4/C5/C6) rings, respectively. The two fused rings in the molecule are inclined at 15.30 (12)°. The thiazine ring in the molecule adopts a half chair conformation which is in accordance with already published data. The r. m. s. deviation for the ring is 0.207 (2) Å. The molecule shows the formation of a six membered S^1^_1_(6) ring motif (Bernstein, *et al.*, 1995) by a O—H···O intramolecular hydrogen bonding interaction between the hydroxyl group in 4-position of the thiazine ring and the carbonyl oxygen atom of the methyl ester substituent. The resulting ring (C7/O1/H1O/O4/C9/C8) deviates from the least square plane with a r. m. s. deviation of 0.052 (4) Å. The maximum deviation is measured for O1 = 0.08 (2) Å and H1O = -0.08 (3) Å. The *N*-butyl moiety shows a maximum deviation of the thiazine ring of about 83.52 (11)° and it is *anti* with respect to the methyl ester.

### Experimental

The synthesis of the title compound has already been published (Arshad *et al.*, 2011*a*). Recrystallization has been performed from a methanolic solution by slow evaporation of the solvent.

### Refinement

Carbon bound H-atoms were positioned in idealized positions with C—H = 0.95 Å, C—H = 0.99 Å and C—H = 0.98 Å for aromatic, methylene and methyl carbon atoms respectively, and were refined using a riding model with *U*_iso_(H) = 1.2 *U*_eq_(C) for aromatic and methylene and *U*_iso_(H) = 1.5 *U*_eq_(C) for methyl carbon atoms. The O–H hydrogen atom was located in the difference map and was refined with O–H= 0.84 (2) Å and *U*_iso_(H) = 1.5 *U*_eq_(O). Electron density synthesis with coefficients *F_o_*—Fc: Highest peaks are 1.70 at 0.3972 0.1429 0.4219 [0.82 Å from N1] & 1.55 at 0.3978 0.1460 0.5612 [0.91 Å from S1] and deepest hole -0.49 at 0.3776 0.1772 0.4612 [0.59 A from S1]. A disorder of the thiazine ring could not be resolved.

The reflections 13 1 1, 1 3 1, -9 7 2 and 11 1 1 for which (I_obs_)-(I_calc_)/σW > 10 were omitted in the final refinement.

### Crystal data

**Table:**

C_14_H_17_NO_5_S	*F*(000) = 1312
*M**_r_* = 311.35	*D*_x_ = 1.414 Mg m^−^^3^
Monoclinic, *C*2/*c*	Mo *K*α radiation, λ = 0.71073 Å
Hall symbol: -C 2yc	Cell parameters from 5544 reflections
*a* = 25.173 (7) Å	θ = 2.3–28.4°
*b* = 9.280 (2) Å	µ = 0.24 mm^−^^1^
*c* = 12.531 (3) Å	*T* = 100 K
β = 91.741 (3)°	Block, colorless
*V* = 2926.0 (13) Å^3^	0.44 × 0.31 × 0.25 mm
*Z* = 8	

### Data collection

**Table:**

Bruker SMART 1K diffractometer	3498 independent reflections
Radiation source: fine-focus sealed tube	3132 reflections with *I* > 2σ(*I*)
Graphite monochromator	*R*_int_ = 0.031
φ and ω scans	θ_max_ = 28.4°, θ_min_ = 2.3°
Absorption correction: multi-scan (*SADABS*; Bruker, 2001)	*h* = −32→33
*T*_min_ = 0.901, *T*_max_ = 0.942	*k* = −12→12
12490 measured reflections	*l* = −16→16

### Refinement

**Table:**

Refinement on *F*^2^	Primary atom site location: structure-invariant direct methods
Least-squares matrix: full	Secondary atom site location: difference Fourier map
*R*[*F*^2^ > 2σ(*F*^2^)] = 0.070	Hydrogen site location: inferred from neighbouring sites
*wR*(*F*^2^) = 0.210	H-atom parameters constrained
*S* = 1.10	*w* = 1/[σ^2^(*F*_o_^2^) + (0.0839*P*)^2^ + 25.4705*P*] where *P* = (*F*_o_^2^ + 2*F*_c_^2^)/3
3498 reflections	(Δ/σ)_max_ < 0.001
193 parameters	Δρ_max_ = 1.70 e Å^−^^3^
0 restraints	Δρ_min_ = −0.49 e Å^−^^3^

### Special details

**Table:**

Geometry. All e.s.d.'s (except the e.s.d. in the dihedral angle between two l.s. planes) are estimated using the full covariance matrix. The cell e.s.d.'s are taken into account individually in the estimation of e.s.d.'s in distances, angles and torsion angles; correlations between e.s.d.'s in cell parameters are only used when they are defined by crystal symmetry. An approximate (isotropic) treatment of cell e.s.d.'s is used for estimating e.s.d.'s involving l.s. planes.
Refinement. Refinement of *F*^2^ against ALL reflections. The weighted *R*-factor *wR* and goodness of fit *S* are based on *F*^2^, conventional *R*-factors *R* are based on *F*, with *F* set to zero for negative *F*^2^. The threshold expression of *F*^2^ > σ(*F*^2^) is used only for calculating *R*-factors(gt) *etc*. and is not relevant to the choice of reflections for refinement. *R*-factors based on *F*^2^ are statistically about twice as large as those based on *F*, and *R*- factors based on ALL data will be even larger.

### Fractional atomic coordinates and isotropic or equivalent isotropic displacement parameters (Å^2^)

**Table:**

	*x*	*y*	*z*	*U*_iso_*/*U*_eq_	
S1	0.39425 (3)	0.14891 (9)	0.48843 (6)	0.0157 (2)	
O1	0.28629 (10)	0.2661 (3)	0.23550 (19)	0.0204 (5)	
H1O	0.3009	0.2796	0.1770	0.024*	
O2	0.39702 (10)	0.3001 (3)	0.5114 (2)	0.0212 (5)	
O3	0.42548 (10)	0.0508 (3)	0.55284 (19)	0.0218 (5)	
O4	0.36673 (10)	0.3043 (3)	0.11802 (19)	0.0222 (5)	
O5	0.44802 (10)	0.2584 (3)	0.19007 (19)	0.0202 (5)	
N1	0.40931 (11)	0.1252 (3)	0.3631 (2)	0.0166 (6)	
C1	0.32710 (13)	0.0977 (4)	0.4870 (3)	0.0164 (6)	
C2	0.30543 (14)	0.0261 (4)	0.5729 (3)	0.0206 (7)	
H2	0.3273	−0.0019	0.6325	0.025*	
C3	0.25114 (15)	−0.0039 (4)	0.5702 (3)	0.0231 (7)	
H3	0.2358	−0.0522	0.6286	0.028*	
C4	0.21938 (14)	0.0363 (4)	0.4828 (3)	0.0215 (7)	
H4	0.1824	0.0160	0.4818	0.026*	
C5	0.24152 (13)	0.1063 (4)	0.3964 (3)	0.0191 (7)	
H5	0.2195	0.1339	0.3370	0.023*	
C6	0.29595 (13)	0.1362 (4)	0.3967 (3)	0.0173 (6)	
C7	0.32050 (13)	0.2003 (4)	0.3037 (3)	0.0171 (6)	
C8	0.37370 (13)	0.1909 (4)	0.2873 (2)	0.0167 (6)	
C9	0.39560 (13)	0.2557 (3)	0.1903 (3)	0.0169 (6)	
C10	0.47078 (15)	0.3216 (5)	0.0957 (3)	0.0273 (8)	
H10A	0.4646	0.2573	0.0346	0.041*	
H10B	0.5091	0.3351	0.1081	0.041*	
H10C	0.4540	0.4151	0.0809	0.041*	
C11	0.43520 (13)	−0.0124 (4)	0.3326 (3)	0.0182 (6)	
H11A	0.4666	−0.0282	0.3805	0.022*	
H11B	0.4480	−0.0028	0.2590	0.022*	
C12	0.39938 (14)	−0.1447 (4)	0.3374 (3)	0.0195 (7)	
H12A	0.3675	−0.1300	0.2906	0.023*	
H12B	0.3876	−0.1584	0.4114	0.023*	
C13	0.42921 (15)	−0.2787 (4)	0.3015 (3)	0.0227 (7)	
H13A	0.4631	−0.2864	0.3432	0.027*	
H13B	0.4378	−0.2683	0.2253	0.027*	
C14	0.39715 (17)	−0.4169 (4)	0.3160 (4)	0.0340 (9)	
H14A	0.3861	−0.4238	0.3902	0.051*	
H14B	0.4191	−0.5004	0.2989	0.051*	
H14C	0.3656	−0.4149	0.2682	0.051*	

### Atomic displacement parameters (Å^2^)

**Table:**

	*U*^11^	*U*^22^	*U*^33^	*U*^12^	*U*^13^	*U*^23^
S1	0.0160 (4)	0.0161 (4)	0.0148 (4)	0.0010 (3)	−0.0017 (3)	−0.0007 (3)
O1	0.0192 (12)	0.0225 (12)	0.0192 (12)	0.0038 (9)	−0.0023 (9)	0.0033 (9)
O2	0.0222 (12)	0.0166 (12)	0.0244 (12)	−0.0010 (9)	−0.0021 (9)	−0.0047 (9)
O3	0.0210 (12)	0.0254 (13)	0.0188 (11)	0.0036 (10)	−0.0032 (9)	0.0026 (9)
O4	0.0236 (13)	0.0227 (13)	0.0201 (12)	0.0006 (10)	−0.0020 (9)	0.0049 (9)
O5	0.0202 (12)	0.0226 (12)	0.0176 (11)	−0.0026 (9)	0.0000 (9)	0.0032 (9)
N1	0.0200 (14)	0.0180 (13)	0.0116 (12)	0.0010 (11)	−0.0013 (10)	0.0003 (10)
C1	0.0149 (14)	0.0163 (15)	0.0180 (15)	0.0021 (12)	−0.0008 (11)	−0.0025 (12)
C2	0.0224 (17)	0.0195 (16)	0.0199 (16)	0.0047 (13)	0.0011 (12)	0.0014 (12)
C3	0.0277 (18)	0.0184 (16)	0.0235 (17)	0.0050 (14)	0.0066 (13)	0.0026 (13)
C4	0.0177 (16)	0.0179 (16)	0.0290 (18)	−0.0012 (12)	0.0031 (13)	−0.0043 (13)
C5	0.0173 (15)	0.0169 (15)	0.0232 (16)	0.0008 (12)	−0.0005 (12)	−0.0033 (12)
C6	0.0193 (16)	0.0151 (15)	0.0175 (15)	0.0026 (12)	−0.0005 (11)	−0.0023 (11)
C7	0.0205 (16)	0.0142 (15)	0.0164 (15)	0.0020 (12)	−0.0037 (11)	−0.0017 (11)
C8	0.0208 (16)	0.0149 (14)	0.0142 (14)	0.0000 (12)	−0.0023 (11)	0.0006 (11)
C9	0.0199 (16)	0.0110 (14)	0.0197 (15)	−0.0006 (11)	−0.0008 (12)	−0.0005 (11)
C10	0.0236 (18)	0.036 (2)	0.0225 (17)	−0.0062 (16)	0.0038 (13)	0.0081 (15)
C11	0.0180 (15)	0.0179 (15)	0.0187 (15)	0.0017 (12)	0.0008 (11)	0.0009 (12)
C12	0.0215 (16)	0.0174 (16)	0.0196 (16)	0.0031 (12)	−0.0002 (12)	−0.0007 (12)
C13	0.0281 (18)	0.0206 (17)	0.0193 (16)	0.0060 (14)	0.0007 (13)	−0.0014 (13)
C14	0.034 (2)	0.0225 (19)	0.046 (2)	0.0012 (16)	−0.0062 (17)	−0.0043 (17)

### Geometric parameters (Å, º)

**Table:**

S1—O2	1.433 (3)	C5—H5	0.9500
S1—O3	1.435 (2)	C6—C7	1.462 (5)
S1—N1	1.642 (3)	C7—C8	1.364 (5)
S1—C1	1.756 (3)	C8—C9	1.478 (5)
O1—C7	1.341 (4)	C10—H10A	0.9800
O1—H1O	0.8400	C10—H10B	0.9800
O4—C9	1.230 (4)	C10—H10C	0.9800
O5—C9	1.320 (4)	C11—C12	1.526 (5)
O5—C10	1.453 (4)	C11—H11A	0.9900
N1—C8	1.423 (4)	C11—H11B	0.9900
N1—C11	1.489 (4)	C12—C13	1.527 (5)
C1—C2	1.390 (5)	C12—H12A	0.9900
C1—C6	1.404 (4)	C12—H12B	0.9900
C2—C3	1.394 (5)	C13—C14	1.528 (6)
C2—H2	0.9500	C13—H13A	0.9900
C3—C4	1.388 (5)	C13—H13B	0.9900
C3—H3	0.9500	C14—H14A	0.9800
C4—C5	1.393 (5)	C14—H14B	0.9800
C4—H4	0.9500	C14—H14C	0.9800
C5—C6	1.398 (5)		
			
O2—S1—O3	119.06 (15)	N1—C8—C9	118.7 (3)
O2—S1—N1	108.17 (15)	O4—C9—O5	124.0 (3)
O3—S1—N1	108.32 (15)	O4—C9—C8	121.9 (3)
O2—S1—C1	107.95 (15)	O5—C9—C8	114.1 (3)
O3—S1—C1	110.17 (16)	O5—C10—H10A	109.5
N1—S1—C1	101.78 (15)	O5—C10—H10B	109.5
C7—O1—H1O	109.5	H10A—C10—H10B	109.5
C9—O5—C10	115.4 (3)	O5—C10—H10C	109.5
C8—N1—C11	117.9 (3)	H10A—C10—H10C	109.5
C8—N1—S1	114.9 (2)	H10B—C10—H10C	109.5
C11—N1—S1	118.5 (2)	N1—C11—C12	114.6 (3)
C2—C1—C6	121.6 (3)	N1—C11—H11A	108.6
C2—C1—S1	121.5 (2)	C12—C11—H11A	108.6
C6—C1—S1	117.0 (3)	N1—C11—H11B	108.6
C1—C2—C3	119.0 (3)	C12—C11—H11B	108.6
C1—C2—H2	120.5	H11A—C11—H11B	107.6
C3—C2—H2	120.5	C11—C12—C13	110.3 (3)
C4—C3—C2	120.4 (3)	C11—C12—H12A	109.6
C4—C3—H3	119.8	C13—C12—H12A	109.6
C2—C3—H3	119.8	C11—C12—H12B	109.6
C3—C4—C5	120.3 (3)	C13—C12—H12B	109.6
C3—C4—H4	119.9	H12A—C12—H12B	108.1
C5—C4—H4	119.9	C12—C13—C14	112.4 (3)
C4—C5—C6	120.4 (3)	C12—C13—H13A	109.1
C4—C5—H5	119.8	C14—C13—H13A	109.1
C6—C5—H5	119.8	C12—C13—H13B	109.1
C5—C6—C1	118.3 (3)	C14—C13—H13B	109.1
C5—C6—C7	121.1 (3)	H13A—C13—H13B	107.8
C1—C6—C7	120.5 (3)	C13—C14—H14A	109.5
O1—C7—C8	123.2 (3)	C13—C14—H14B	109.5
O1—C7—C6	114.4 (3)	H14A—C14—H14B	109.5
C8—C7—C6	122.3 (3)	C13—C14—H14C	109.5
C7—C8—N1	121.9 (3)	H14A—C14—H14C	109.5
C7—C8—C9	119.3 (3)	H14B—C14—H14C	109.5
			
O2—S1—N1—C8	−63.0 (3)	C5—C6—C7—O1	19.3 (5)
O3—S1—N1—C8	166.7 (2)	C1—C6—C7—O1	−163.4 (3)
C1—S1—N1—C8	50.6 (3)	C5—C6—C7—C8	−159.5 (3)
O2—S1—N1—C11	150.0 (2)	C1—C6—C7—C8	17.9 (5)
O3—S1—N1—C11	19.7 (3)	O1—C7—C8—N1	177.6 (3)
C1—S1—N1—C11	−96.5 (3)	C6—C7—C8—N1	−3.8 (5)
O2—S1—C1—C2	−102.1 (3)	O1—C7—C8—C9	0.1 (5)
O3—S1—C1—C2	29.5 (3)	C6—C7—C8—C9	178.7 (3)
N1—S1—C1—C2	144.2 (3)	C11—N1—C8—C7	112.4 (4)
O2—S1—C1—C6	76.0 (3)	S1—N1—C8—C7	−34.9 (4)
O3—S1—C1—C6	−152.4 (2)	C11—N1—C8—C9	−70.1 (4)
N1—S1—C1—C6	−37.7 (3)	S1—N1—C8—C9	142.7 (3)
C6—C1—C2—C3	−2.0 (5)	C10—O5—C9—O4	−1.1 (5)
S1—C1—C2—C3	176.0 (3)	C10—O5—C9—C8	−179.9 (3)
C1—C2—C3—C4	0.4 (5)	C7—C8—C9—O4	−8.0 (5)
C2—C3—C4—C5	0.4 (5)	N1—C8—C9—O4	174.3 (3)
C3—C4—C5—C6	0.3 (5)	C7—C8—C9—O5	170.8 (3)
C4—C5—C6—C1	−1.8 (5)	N1—C8—C9—O5	−6.8 (4)
C4—C5—C6—C7	175.6 (3)	C8—N1—C11—C12	−76.7 (4)
C2—C1—C6—C5	2.7 (5)	S1—N1—C11—C12	69.3 (3)
S1—C1—C6—C5	−175.4 (3)	N1—C11—C12—C13	178.3 (3)
C2—C1—C6—C7	−174.8 (3)	C11—C12—C13—C14	174.0 (3)
S1—C1—C6—C7	7.1 (4)		

### Hydrogen-bond geometry (Å, º)

**Table:**

*D*—H···*A*	*D*—H	H···*A*	*D*···*A*	*D*—H···*A*
O1—H1*O*···O4	0.84	1.85	2.564 (4)	142

## References

[bb1] Arshad, M. N., Khan, I. U., Zia-ur-Rehman, M., Danish, M. & Holman, K. T. (2011*b*). *Acta Cryst.* E**67**, o3445. 10.1107/S160053681104966X PMC3239077 22199925

[bb2] Arshad, M. N., Khan, I. U., Zia-ur-Rehman, M. & Shafiq, M. (2011*a*). *Asian J. Chem.* **23**, o2801–2805.

[bb3] Arshad, M. N., Zia-ur-Rehman, M., Khan, I. U., Mustafa, G., Shafiq, M., Rafique, H. M. & Holman, K. T. (2012). *Walailak J. Sci. Tech.* **10** In the press.

[bb4] Barbour, L. J. (2001). *J. Supramol. Chem.* **1**, 189–191.

[bb5] Bernstein, J., Davis, R. E., Shimoni, L. & Chang, N.-L. (1995). *Angew. Chem. Int. Ed. Engl.* **34**, 1555–1573.

[bb6] Bruker (2001). *SADABS*, *APEX2* and *SAINT* Bruker AXS Inc., Madison, Wisconsin, USA.

[bb7] Cremer, D. & Pople, J. A. (1975). *J. Am. Chem. Soc.* **97**, 1354–1358.

[bb8] Farrugia, L. J. (1999). *J. Appl. Cryst.* **32**, 837–838.

[bb9] Sheldrick, G. M. (2008). *Acta Cryst.* A**64**, 112–122. 10.1107/S0108767307043930 18156677

[bb10] Spek, A. L. (2009). *Acta Cryst.* D**65**, 148–155. 10.1107/S090744490804362X PMC2631630 19171970

[bb11] Zia-ur-Rehman, M., Anwar, J., Ahmad, S. & Siddiqui, H. L. (2006). *Chem. Pharm. Bull.* **54**, 1175–1178. 10.1248/cpb.54.1175 16880664

